# Intravenously administered iron oxide nanoparticles with different coatings reversibly perturb immune cells in peripheral blood without inducing toxicity in mice

**DOI:** 10.3389/ftox.2025.1673416

**Published:** 2025-10-14

**Authors:** Preethi Korangath, Chun-Ting Yang, Sean Healy, Cordula Grüttner, Kathleen Gabrielson, Robert Ivkov

**Affiliations:** ^1^ Department of Radiation Oncology and Molecular Radiation Sciences, School of Medicine, Johns Hopkins University, Baltimore, MD, United States; ^2^ Micromod Partikeltechnologie GmbH, Rostock, Germany; ^3^ Department of Molecular and Comparative Pathobiology, School of Medicine, Johns Hopkins University, Baltimore, MD, United States; ^4^ Department of Mechanical Engineering, Whiting School of Engineering, Johns Hopkins University, Baltimore, MD, United States; ^5^ Department of Oncology, Sidney Kimmel Comprehensive Cancer Centre, School of Medicine, Johns Hopkins University, Baltimore, MD, United States; ^6^ Department of Materials Science and Engineering, Whiting School of Engineering, Johns Hopkins University, Baltimore, MD, United States; ^7^ Institute for NanoBioTechnology, Whiting School of Engineering, Johns Hopkins University, Baltimore, MD, United States

**Keywords:** iron oxide nanoparticles, immunotoxicity, biodistribution, PEGylated bionized nanoferrite nanoparticles, Micromer^®^, toxicity, Venofer

## Abstract

**Introduction:**

Iron oxide nanoparticle formulations are widely used in clinical applications and have recently been explored for hyperthermia therapy, cancer imaging and treatment. Here, we report the effects of intravenously injected pegylated or poly acrylic acid decorated iron oxide nanoparticles coated with hydroxyethyl starch (HES) on host immune system and organs. These particles were compared with sucrose coated iron oxide nanoparticle (Venofer^®^) and the coating compound HES–both FDA approved agents–alongside non-iron oxide polystyrene nanoparticles coated with HES (micromer^®^).

**Methods:**

Toxicity analysis was performed in healthy female normal FVB/NJ mice 60 days after nanoparticle injection, with complete blood analysis conducted at multiple time-points. In a separate cohort, nanoparticle biodistribution 24 h post-intravenous injection was evaluated using a HER2 overexpressing breast cancer mouse model.

**Results:**

Toxicity analysis revealed no adverse effects on liver or kidneys with any of the tested formulations after 60 days. Immune cell perturbations were observed at early time points following iron oxide nanoparticle injection but normalized by the study endpoint. Biodistribution analysis demonstrated that the nanoparticle coating dictated their accumulation across various organs, with significant tumor accumulation observed for pegylated iron oxide nanoparticles and Venofer^®^.

**Conclusion:**

Iron oxide nanoparticle formulations exert a transient effect on the host immune system and some exhibit tumor accumulation, suggesting their potential for further development in cancer imaging and treatment.

## 1 Introduction

Nanoparticles have a wide range of applications in different fields (Bayda et al., 2019). In the biomedical field, their properties are explored for use in safe and effective ways. One of the oldest FDA-approved nanoparticle formulations, iron oxide, is used in clinical applications for the treatment of anemia and as an MRI contrast agent (Dadfar et al., 2019; Iv et al., 2015; Alphandéry, 2020; Auerbach et al., 2021; Kobayashi, 2011). Apart from this, their unique ability to generate heat under an alternating magnetic field is currently being investigated for cancer hyperthermia treatment (Attaluri et al., 2020; Attaluri et al., 2015; Oei et al., 2019; Shirvalilou et al., 2021; Rodriguez et al., 2024). Although a handful of iron oxide nanoparticle formulations have been used clinically for decades (Gafter-Gvili et al., 2014; Lebrun et al., 2017; Gemici et al., 2016), concerns persist about different new formulations with varying coating and surface properties. The physical characteristics of nanoparticle formulations enable different biodistribution kinetics, and hence, the toxicity profile could change drastically and should be monitored (Sharma et al., 2018; Cole et al., 2011; Nowak-Jary and Machnicka, 2023).

In our previous study, we found that hydroxyethyl starch (HES)-coated bionized nanoferrite (BNF–starch) nanoparticle (BP), human epidermal growth factor receptor 2 (HER2+ve) tumor-targeting antibody-coated BP (BNF–starch–herceptin ((BH)), and a non-specific control antibody-coated BP, (BNF–starch–IgG (BIgG)) were taken up by stromal and immune cells in the tumor microenvironment (TME) after systemic injection (Healy et al., 2023). Mice injected with BP nanoparticles exhibited delayed tumor growth and increased infiltration of immune cells into the tumors in immune-competent mice (Korangath et al., 2020). Further mechanistic studies with BP nanoparticles revealed the activation of Toll-like receptor 3 (TLR3)-mediated innate-to-adaptive immune signaling as the mechanism of tumor growth and metastasis inhibition in multiple mouse breast cancer models, with no discernible toxicity observed in the mice (Korangath et al., 2024). Recently, we reported organ-specific activation of dendritic cells by BP after systemic injection in normal mice (Song et al., 2024).

This prior knowledge led us to investigate the effects of varying coatings and cores of nanoparticle constructs for their anti-tumor effects. Before doing so, we aimed to evaluate their toxicity profile in healthy mice and their biodistribution in tumor-bearing mice. We also hypothesized that the core of the nanoparticle construct may influence the host immune system. For this, we performed toxicity analysis and organ distribution of two iron oxide nanoparticles: 1) PEGylated BNF–starch nanoparticles (PEG-BP) that contain an iron oxide core coated with hydroxyethyl starch (such as BP) and polyethylene glycol (PEG) on the surface to increase circulation time in the blood and 2) poly acrylic acid (PAA)-coated BNF nanoparticles (BNF–PAA) that have an iron oxide core similar to BP with PAA coating. We compared these nanoparticles with a) an FDA-approved clinically available iron-sucrose nanoparticle, Venofer^®^ (VeFe), b) the coating material, HES, and c) a non-magnetic nanoparticle type, Micromer^®^, which is a polystyrene nanoparticle with HES coating. HES is widely used in clinics to expand the blood volume for transfusions (Myburgh et al., 2012). This comparative analysis was performed to delineate the effect of coating or the core on the host system when intravenously administered.

## 2 Materials and methods

### 2.1 Nanoparticles

Micromod Partikeltechnologie GmbH, Rostock, Germany, supplied HES, Micromer^®^ with HES, PEG-BP, and BNF–PAA. The clinical formulation of iron sucrose (Venofer^®^) was purchased from the Johns Hopkins Pharmacy, Baltimore, MD, United States.

#### 2.1.1 Synthesis of BNF–starch PEG 25.000-OMe

A total of 100 mg MeO-PEG-NH_2_ (MW: 25.000 Da, IRIS Biotech GmbH, Germany) was dissolved in 10 mL of borate buffer at pH = 9.0 and added to 10 mL of 100 nm BNF–starch particles with epoxy groups on the surface (10-08-102, Micromod Partikeltechnologie GmbH, Germany, 20 mg/mL). The suspension was shaken for 16 h at ambient temperature. After magnetic separation using LifeSep 50SX (Dexter Magnetic Technologies, United States), the supernatant was decanted, and the sediment was suspended in 10 mL water. The washing step was repeated two additional times. Finally, the suspension was filtered through a 0.22 µm syringe filter (PES, Millipore, United States).

#### 2.1.2 Synthesis of BNF-PAA-Na

A measure of 6.8 g of polyacrylic acid sodium salt (MW: 1,200 Da, 45% (v/v) in water, Sigma-Aldrich Chemie GmbH, Germany) was diluted with 12 mL of distilled water and added to 23 mL of iron oxide suspension (83-00-402, Micromod Partikeltechnologie GmbH, Germany, 20 mg/mL). The suspension was homogenized at 80 °C and 1,000 bar (M110Y Microfluidizer^®^, Microfluidics, United States) for 20 min. After cooling to ambient temperature, the particles were separated using LifeSep 50SX (Dexter Magnetic Technologies, United States), the supernatant was decanted, and the sediment was suspended in 10 mL of distilled water. The washing step was repeated two additional times. Finally, the suspension was filtered through a 0.22-µm syringe filter (PES, Millipore, United States). Micromer^®^ nanoparticle synthesis has been reported previously (Korangath et al., 2024). The measured physical characteristics of the nanoparticles are provided in Supplementary Table S1.

### 2.2 Animals

Female FVB/NJ mice (8 weeks old) were purchased from Jackson labs, ME, United States. The Institutional Animal Care and Use Committee (IACUC) at Johns Hopkins University approved all animal experiments. Female mice were selected due to the breast tumor model used in this study. Mice were fed a normal diet and water *ad libitum*. Mice were maintained under a standard 12-h of light/dark cycle. At the experimental endpoints, as described below, all mice were sacrificed by carbon dioxide inhalation in a controlled flow rate chamber as per the IACUC recommendations.

### 2.3 Experimental design

This study was conducted along with a previously reported toxicity and biodistribution analysis of BP, and hence, the control PBS-treated mice were the same as reported earlier (Korangath et al., 2024).

#### 2.3.1 Toxicity study

Upon arrival, the mice were acclimatized for 1 week. After 1 week, they were randomly divided into groups of eight mice each. Each group received the following treatment through tail-vein injection, including phosphate-buffered saline (PBS), VeFe (Venofer^®^, 5 mg Fe/mouse), HES (3 mg/mouse), Micromer^®^ (3 mg/mouse), PEG-BP (5 mg Fe/mouse), and BNF–PAA (5 mg Fe/mouse). The concentration was determined based on our previous study (Korangath et al., 2024), where BNF–starch (BP) nanoparticles showed a therapeutic effect at 5 mg Fe/mouse. The concentrations of HES and Micromer^®^ were equivalent to the starch concentration on PEG-BP nanoparticles. The body weights of mice were recorded before injection and, thereafter, weekly for 8 weeks.

#### 2.3.2 Biodistribution study

After 1 week of acclimatization, the mice were transplanted with HuHER2 allograft derived from HuHER2 transgenic mice, as described before (Korangath et al., 2020). When the tumor volume reached approximately 150 mm^3^, they were randomly assigned to groups of five mice each and received treatment as mentioned above through tail-vein injection. After 24 h of injection, mice were sacrificed to collect blood and tissues for further analysis.

### 2.4 Blood analysis

The whole-blood was collected from the facial vein for a toxicity study on days 1, 3, 8, 15, 30, and 60 after injection. For the biodistribution study, blood was collected from the heart after 24 h of nanoparticle injection. Blood collected in EDTA-coated tubes (BD Microtainer^®^ Tube with K2EDTA, BD Biosciences, San Jose, CA) was analyzed for complete blood count in an automated CBC analyzer (ProCyte Dx TM, IDEXX, Westbrook, Maine). Alternate mice (4 mice/time-point) from each group were used on days 1, 3, and 8 days to minimize stress. At the endpoint, all mice were sacrificed to collect blood from the heart. Serum was used for liver function [alkaline phosphatase (ALP), alanine transferase (ALT), and aspartate transaminase (AST) activities] and kidney function [creatinine and blood urea nitrogen levels (BUN)] analyses using an automated clinical chemistry analyzer (Vet Ace^TM^, Alfa Wassermann, West Caldwell, NJ).

### 2.5 Inductively coupled plasma mass spectroscopy

Serum and one-half of the tumor, liver, spleen, lungs, kidneys, and lymph node were collected 24 h after injection and lyophilized. The dry weights of the tissues were recorded, and a known amount of tissue was analyzed for absolute iron content by inductively coupled plasma mass spectroscopy (ICP-MS), as described before (Korangath et al., 2020).

### 2.6 Histopathology

After 24 h (biodistribution cohort) or 60 days (toxicity cohort) following treatment, all mice were sacrificed to collect the tumor (biodistribution cohort), liver, lungs, spleen, sternum (for bone marrow), kidneys, adrenal glands, pancreas, inguinal lymph nodes, heart, and intestine. From the biodistribution cohort, one-half of the tissues were used for histology analysis, and the other half was used for ICP-MS, as described above. All tissues were fixed in 10% formalin for 48 h. After 48 h, tissues were embedded in paraffin blocks and sectioned for slide preparation. The slides were then stained with hematoxylin and eosin (H&E) and Prussian blue (PB) for visualizing tissue morphology and iron oxide nanoparticle distribution, respectively. All Prussian blue slides from the toxicity cohort were digitized in an automated image scanner, the Aperio ScanScope AT or CS system (Aperio, Vista, CA) at ×40 magnification. All H&E slides from the toxicity cohort were manually evaluated by a veterinary pathologist (KG) to report any adverse effects observed microscopically in any tissue. Liver tissue samples were digitized using a Zeiss scanner. These images were then used to quantify the inflammatory foci present in the liver tissues.

### 2.7 Quantification of inflammatory foci and Prussian blue-positive areas

An equally sized single lobe of liver was quantified manually by counting the number of inflammatory foci present in that lobe from the digitized histology slides. Prussian blue slides were quantified as described before (Korangath et al., 2024).

### 2.8 Statistical analysis

All statistical analyses were conducted using GraphPad Prism software (San Diego, CA). All data are presented as scatter plots with the median. An unpaired, non-parametric Mann–Whitney test was used to compare the difference between the control PBS and treatment groups, with *p* < 0.05 being considered a significant change.

## 3 Results

### 3.1 Overall health and body weight showed no signs of noticeable toxicity with systemic injection of nanoparticles

**FIGURE 1:**
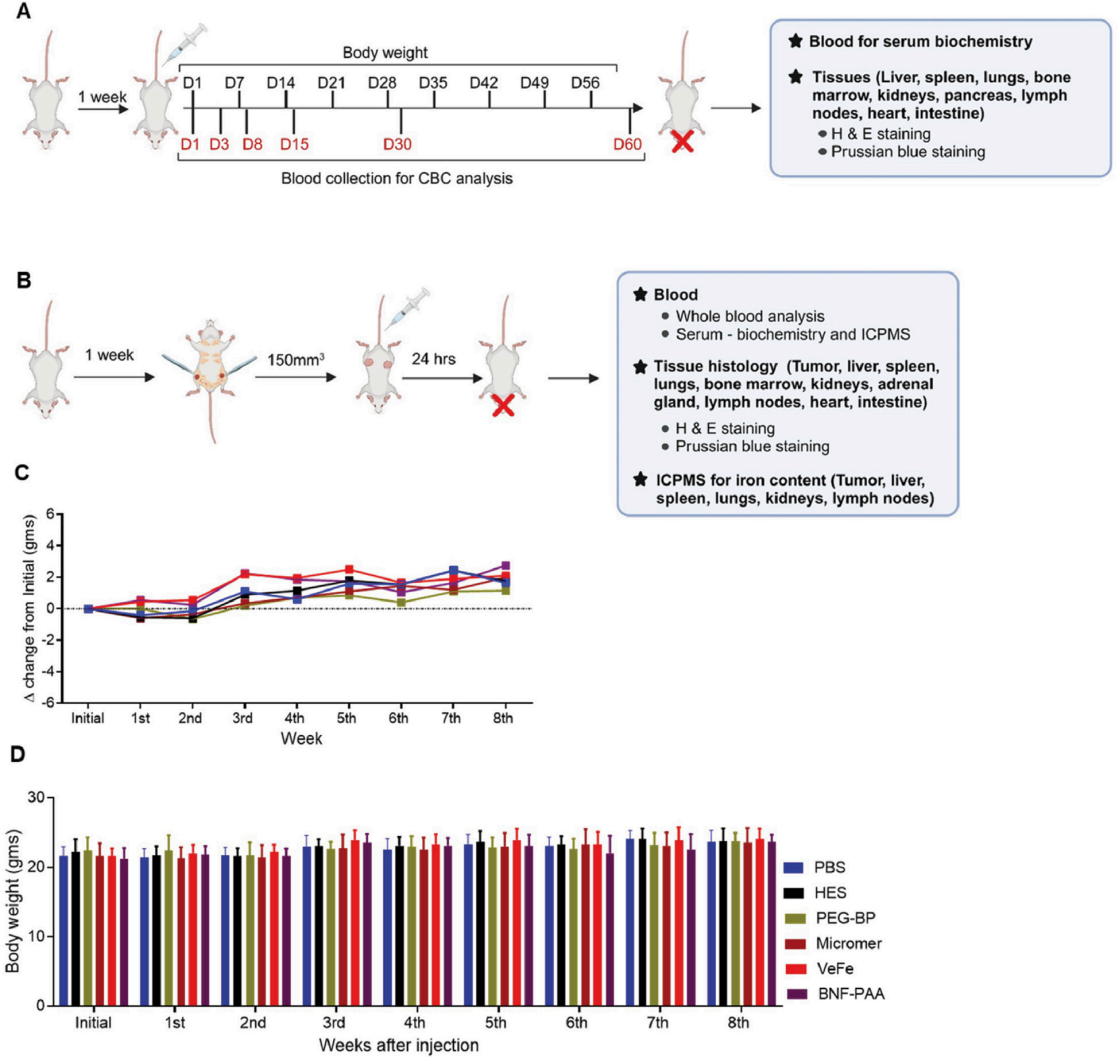
Mice appeared normal with nanoparticle injection. **(A, B)** Overall study design: female FVB/NJ mice (8 weeks old) were acclimatized for 1 week after arrival and proceeded for short-term nanoparticle toxicity study or biodistribution study, as described in Methods. **(C, D)** Measurement of the body weight of mice over 60 days in the toxicity study showed a gradual increase over time without any significant difference between any of the groups.

A schema of the toxicity and biodistribution study design is given in Figures 1A, B. All mice, irrespective of the treatment received, gained weight over time (Figures 1C, D), which indicates a generally good health condition. No adverse behavioral or toxicity changes, such as decreased activity, hunched posture, piloerection/unkempt hair coat, abnormal ocular appearance indicative of corneal ulceration, impaired blinking, or weight loss, were observed in any treatment group throughout the study period (60 days). To further assess additional parameters, we performed hematological evaluation on different days, along with liver and kidney function tests, as indicated in Figure 1A.

### 3.2 Hematological parameters showed transient changes that normalized by day 60

#### 3.2.1 White blood cells

**FIGURE 2:**
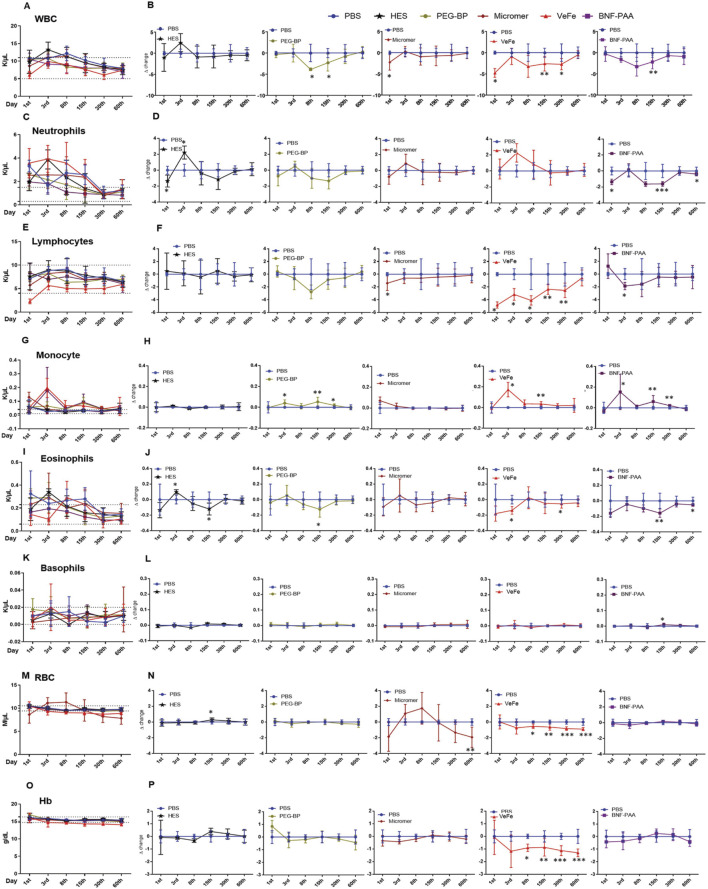
Blood parameters show transient perturbations depending on the nanoparticle formulation. **(A)** Total WBC count at different time-points. Even though significant changes were observed in white blood cell count over time with nanoparticle treatment compared to that in PBS controls, the counts were within the normal range (dotted line, 5–11 K/μL) reported for FVB/NJ mice. **(B)** Differences observed relative to PBS at each time-point for different treatment groups are given as delta change. White blood cells were significantly reduced on day 1 with VeFe and Micromer^®^ treatment. On day 8, the WBC count was significantly lower with PEG-BP treatment. On day 15, a significant decrease was observed in VeFe-, PEG-BP-, and BNF–PAA-treated groups, and on day 30, a significant decrease was observed in the VeFe cohort. It was similar in all the groups by day 60. **(C)** Significant change was observed in neutrophils over time with nanoparticle treatment. Neutrophil count at different time-points is given. Dotted line – 0.3–1.5 K/μL represents the normal range. **(D)** Differences observed relative to PBS at each time-point for different treatment groups are given as the delta change. Neutrophils were significantly decreased on day 1 with HES and BNF-PAA treatment. On day 3, it was significantly increased in the HES group. On days 15 and 60, neutrophils were significantly lower in BNF–PAA-treated mice. **(E)** Significant change in the lymphocyte population over time was observed with nanoparticle treatment. The normal range is from 4 to 10 K/μL. **(F)** Differences observed relative to PBS at each time-point for different treatment groups are given as the delta change. Lymphocytes were significantly decreased on day 1 with VeFe and Micromer^®^ treatment. On day 3, they were significantly decreased in VeFe and BNF–PAA groups. On days 8, 15, and 30, VeFe-treated mice had significantly lower lymphocyte counts. All the values were within the normal range even though a statistically significant change was observed relative to the control except for VeFe on day 1. **(G)** Significant difference was observed in the monocyte population over time with nanoparticle treatment. The normal range for monocytes is from 0.01 K/μL to 0.04 K/μL. **(H)** Differences observed relative to PBS at each time-point for different treatment groups are given as the delta change, which shows deviation from the normal range. Monocytes were significantly increased on day 3 with VeFe, PEG-BP, and BNF–PAA treatment. Similar increase was observed on day 15 for these groups. This increase was persistent on day 30 in mice treated with PEG-BP and BNF–PAA nanoparticles. **(I)** Significant difference was observed in the eosinophil population over time with nanoparticle treatment (normal range: 0.06 K/μL–0.23 K/μL) **(J)** Differences observed relative to PBS at each time-point for different treatment groups showed that eosinophils were significantly decreased with VeFe and increased with HES treatment on day 3. On day 15, a significant decrease in eosinophil count was observed in the HES-, PEG-BP-, and BNF–PAA-treated groups. VeFe-treated mice showed a significant decrease in eosinophil count on day 30. In BNF–PAA-treated mice, a significant decrease was observed even on day 60. Even though a statistically significant change was observed with treatment, all values were within the normal range except for HES on day 3. **(K)** No significant difference was observed in the basophil population over time with nanoparticle treatment in any group at any time-point except with BNF–PAA treatment on day 15. Basophil counts at different time-points are given. The normal range is 0.0 K/μL–0.02 K/μL. **(L)** Difference observed relative to PBS at each-time point for different treatment groups is given as the delta change. **(M)** RBC count at different time-points within the normal range (dotted line 9.4 M/μL–10.5 M/μL) is given. **(N)** Difference observed relative to PBS at each time-point for different treatment groups is given as the delta change. RBC counts were significantly decreased with VeFe treatment from day 8 onward until day 60. On day 15, a significant increase in RBCs was detected in the HES-treated group. On day 60, a significant decrease was observed with Micromer^®^ treatment. Except for VeFe treatment, all changes were within the normal range. **(O)** Changes in hemoglobin (Hb) content over time with nanoparticle treatment at different time points are given along with the normal range (14.7 g/dL–16.3 g/dL). **(P)** Difference observed relative to PBS at each time-point for different treatment groups is given as the delta change, which showed no change except with VeFe treatment. The Hb content was significantly decreased with Fe treatment from day 8 onward until day 60 (Mann–Whitney test/; *p < 0.05, **p < 0.01, and ***p < 0.001).

Whole-blood analysis revealed a transient decrease in white blood cells (WBCs) in the nanoparticle-treated group of mice. Even though there were significant differences, all the values were within the normal limit, and no difference was found on day 60 (Figure 2A). To better understand these differences, we calculated the delta change at each time-point by comparing it to that of the PBS-treated control. As shown in Figure 2B, WBC did not show any change with HES treatment. With PEG-BP treatment, there was a significant decrease in WBCs on days 8 and 15. With Micromer^®^, a significant reduction was observed on day 3, and there was no change thereafter. With VeFe treatment, a significant decrease in WBCs was observed on days 1, 15, and 30. BNF–PAA showed a significant difference from PBS only on day 15 (*p < 0.05 and **p < 0.01). By day 60, no difference was detected in any of the groups.

#### 3.2.2 Neutrophils

There was a difference in the neutrophil count in the whole blood of mice that received treatments. They remained above normal levels for up to 30 days, even in PBS-treated mice (Figure 2C). Compared to PBS, some significant differences were detected in treatment groups on different days (Figure 2D). The neutrophil count was significantly decreased on day 1 with HES and BNF–PAA treatment. On day 3, it was significantly increased in the HES group. On days 15 and 60, neutrophils were significantly lower in the BNF–PAA-treated mice (*p < 0.05 and ***p < 0.001). No change was observed in the VeFe-, Micromer^®^-, or PEG-BP-treated mice.

#### 3.2.3 Lymphocytes

The total lymphocyte counts were within the normal range except for VeFe-treated mice after 24 h, which showed a difference at all time-points until day 30 (Figure 2E). The delta change analysis revealed a significant decrease in lymphocytes on day 1 with VeFe and Micromer^®^ treatment (Figure 2F). On day 3, it was significantly decreased in the VeFe and BNF–PAA groups. On days 8, 15, and 30, VeFe-treated mice had significantly lower lymphocyte counts (*p < 0.05 and **p < 0.005). By day 60, all counts normalized, and no difference was observed.

#### 3.2.4 Monocytes

The monocyte population was significantly increased in peripheral blood initially and was above the normal range in iron oxide nanoparticle-treated cohorts (Figure 2G). This was expected as the monocyte population plays a central role in nanoparticle engulfment. Monocytes were significantly increased on day 3 with VeFe, PEG-BP, and BNF–PAA treatment (Figure 2H). A similar increase was also observed on day 15 in those groups. The increase was persistent on day 30 in mice treated with PEG-BP and BNF–PAA nanoparticles (*p < 0.05 and **p < 0.005). By day 60, the monocyte counts were similar in all groups and were within the normal range.

#### 3.2.5 Eosinophils

Slight changes were detected in the eosinophil count at the initial time-points with different treatments (Figure 2I), but by day 30, all values were within the normal range. Eosinophils significantly decreased with VeFe treatment and increased with HES treatment on day 3 (Figure 2J). On day 15, a significant decrease in eosinophil count was observed in the HES-, PEG-BP-, and BNF–PAA-treated groups. VeFe-treated mice showed a significant decrease in eosinophil count on day 30. BNF–PAA-treated mice showed a significant decrease compared to PBS-treated mice on day 60 (*p < 0.05 and **p < 0.005).

#### 3.2.6 Basophils

No significant difference in the basophil population was observed at any time-point in most of the treatment groups (Figure 2K). Delta change analysis showed a significant decrease with BNF–PAA treatment on day 15 (*p < 0.05), but values remained within the normal range (Figure 2L).

#### 3.2.7 Red blood cells

Interestingly, the red blood cell (RBC) level was slightly lowered by the VeFe and Micromer^®^ treatment (Figure 2M). RBC counts were significantly decreased with VeFe treatment from day 8 onward until day 60 (Figure 2N). On day 15, a significant increase in RBCs was detected in the HES-treated group. On day 60, a significant decrease was observed in Micromer^®^-treated animals (*p < 0.05, **p < 0.005, and ***p < 0.001).

#### 3.2.8 Hemoglobin

Hemoglobin was not changed in any group except in the VeFe-treated mice (Figure 2O). Similar to RBCs, hemoglobin was also significantly lower in the VeFe-treated group of mice from day 8 onward until day 60 (Figure 2P). Although these values were lower, they did not fall below 13.9 g/dL, the threshold usually considered indicative of anemia (*p < 0.05, **p < 0.005, and ***p < 0.001).

### 3.3 Liver and kidney function parameters showed no change on day 60

**FIGURE 3:**
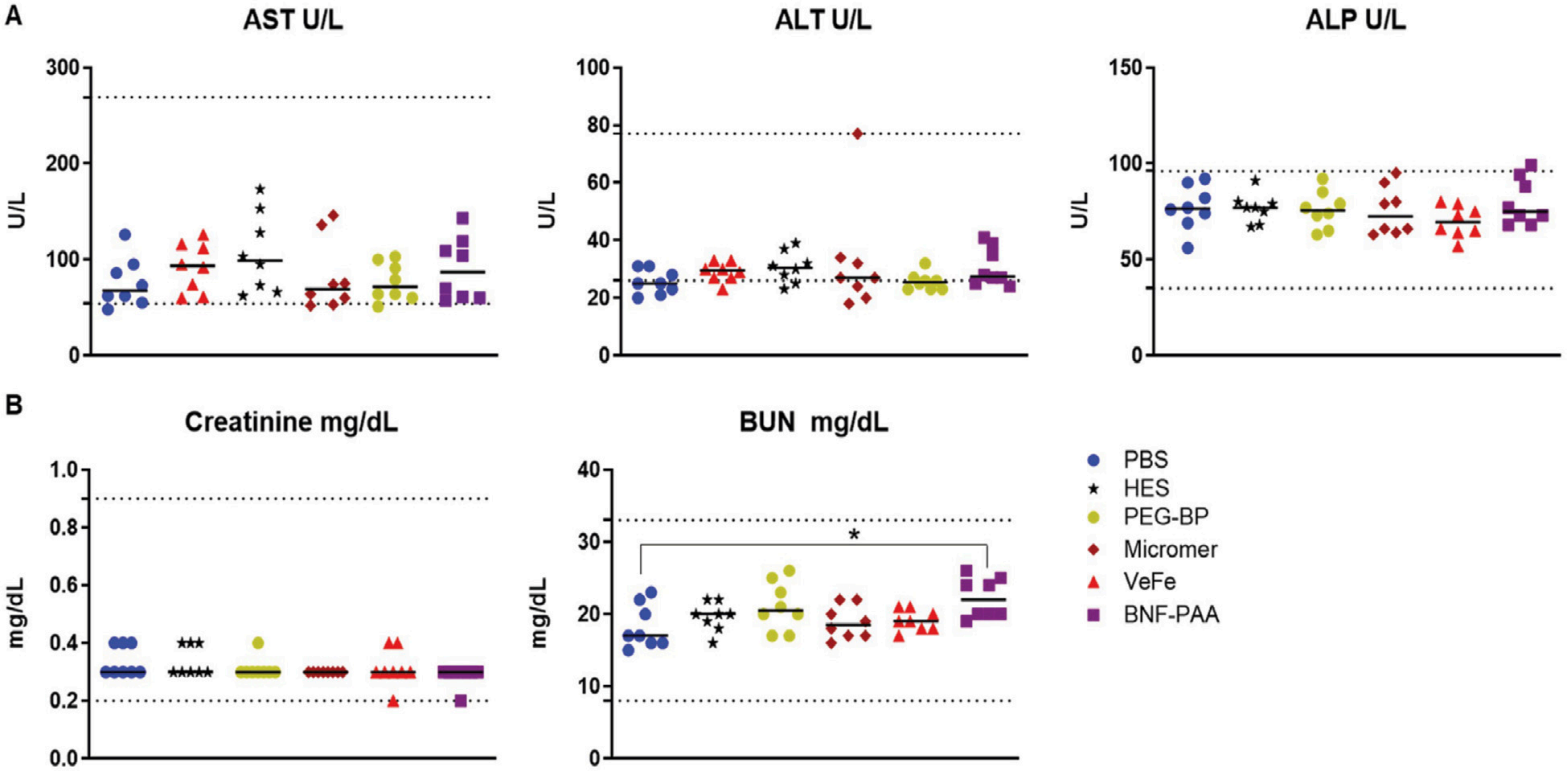
Systemic exposure to a high dose of iron oxide nanoparticles induces no discernible toxicity in healthy mice. No significant differences in liver function or kidney function parameters were observed with nanoparticle treatment. **(A)** Liver function enzymes—aspartate transaminase (AST), alanine transaminase (ALT), or alkaline phosphatase (ALP)—were not significantly changed in any of the treated mice after 60 days of treatment and were within the normal range. Likewise, **(B)** kidney function metabolites—creatinine or blood urea nitrogen (BUN)—were not significantly changed with treatment. Although a statistically significant increase for BUN was detected in the BNF–PAA group relative to that in the control, it was within the normal range (dotted line) (Mann–Whitney test; *p < 0.05).

On day 60, all mice were sacrificed to collect the blood for liver and kidney function analysis. As shown in Figure 3A, no significant difference in liver function enzymes—aspartate transaminase (AST), alanine transaminase (ALT), or alkaline phosphatase (ALP)—was observed in any of the treated mice compared to that in the PBS mice, with all median values within the normal range (dotted lines). Likewise, the kidney function markers—blood urea nitrogen (BUN) and creatinine—were within the normal range for all mice, irrespective of treatment (Figure 3B). Even though within the normal range, the BNF–PAA group showed a significant increase in BUN compared to that in PBS (*p < 0.05). This indicates that the transient changes in hematological parameters did not affect the overall function of the critical organs.

### 3.4 Histopathological analysis showed inflammatory foci in the liver and the accumulation of iron in various organs, with no indication of toxicity with nanoparticle treatment

H&E-stained slides from the liver, lung, heart, pancreas, spleen, kidneys, bone marrow in the sternum, inguinal lymph nodes, and intestine were manually evaluated and examined for toxicity-associated changes. Overall, no distinct or consistent toxicity-associated changes were observed in any of the groups. A small number of inflammatory foci with iron accumulation were observed in VeFe-, PEG-BP-, and BNF–PAA-treated groups. These were considered microgranulomas and quantified separately. Some PEG-BP-treated mice (five out of eight) had minimal inflammation in their lungs, with neutrophil involvement. They were not associated with iron oxide nanoparticles. Apart from these minimal changes, no organ toxicity was observed in any of the organs that were tested. Representative H&E images of each organ from each treatment group are provided in Supplementary Figures S1–S9.

#### 3.4.1 Inflammatory foci

**FIGURE 4:**
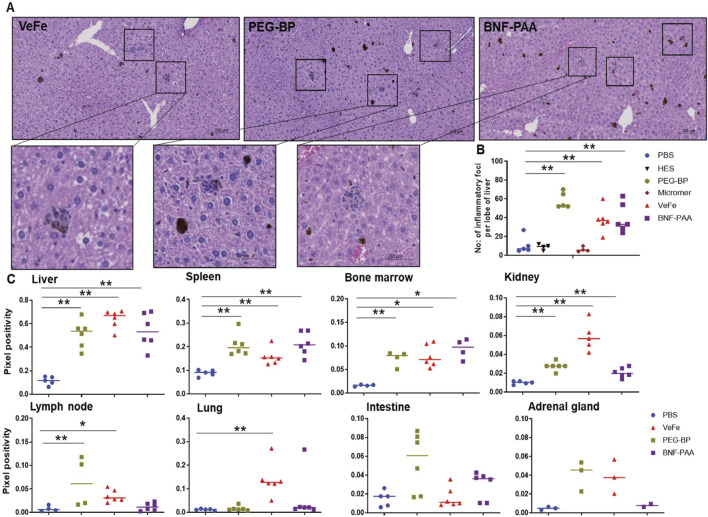
Inflammatory foci were detected in the liver of iron-containing nanoparticle-treated mice with the presence of iron in various organs. A significant increase in the number of inflammatory foci in the liver was identified in all iron-containing formulation-treated mice. **(A)** Representative images of inflammatory foci in the liver associated with nanoparticles from different groups. **(B)** Quantitative analysis showed a significant increase in their number in iron oxide nanoparticle-treated groups. Control PBS-, HES-, and Micromer^®^-treated groups had only a minimal number. **(C)** Quantitative analysis of Prussian blue-stained tissues from the organs of iron oxide nanoparticle-treated mice showed their presence in the organs at varying levels after 60 days of injection.

Upon H&E evaluation, we observed an increase in liver inflammatory foci in some of the treated mice. These were associated with iron oxide nanoparticles (Figure 4A). To quantify this, we digitized the slides and manually counted the number of inflammatory foci from one lobe of the liver from 4–6 mice per group. As shown in Figure 4B, there is a significant increase in the number of inflammatory foci in the livers of VeFe-, PEG-BP-, and BNF–PAA-treated mice compared to that in mice with PBS treatment alone. Both HES and Micromer^®^ showed minimal inflammatory foci and were comparable to those found in PBS-treated mice.

#### 3.4.2 Prussian blue quantification in tissues

Prussian blue positivity as a measure of nanoparticle content was analyzed in tissue slides stained with Prussian blue from different organs. Iron was primarily accumulated in the liver, spleen, bone marrow, and kidneys of all mice treated with iron oxide nanoparticles. We quantified the Prussian blue-positive area in iron oxide nanoparticle-treated mouse tissue and compared it to that of PBS-treated mice (Figure 4C). The liver, spleen, bone marrow, and kidneys had significantly higher Prussian blue positivity, indicating nanoparticle presence even after 60 days of injection. Both PEG-BP- and VeFe-treated mice had significantly higher positivity in the lymph nodes. VeFe-treated mice had significantly higher iron accumulation in the lungs. None of the other cohorts showed iron accumulation in the lungs. The intestine and adrenal gland showed a trend of having higher iron content with PEG-BP treatment, but it was not statistically significant. VeFe-treated mice also showed a statistically insignificant increase in iron accumulation in the adrenal gland.

### 3.5 Nanoparticle composition affects the biodistribution of iron oxide nanoparticles

**FIGURE 5:**
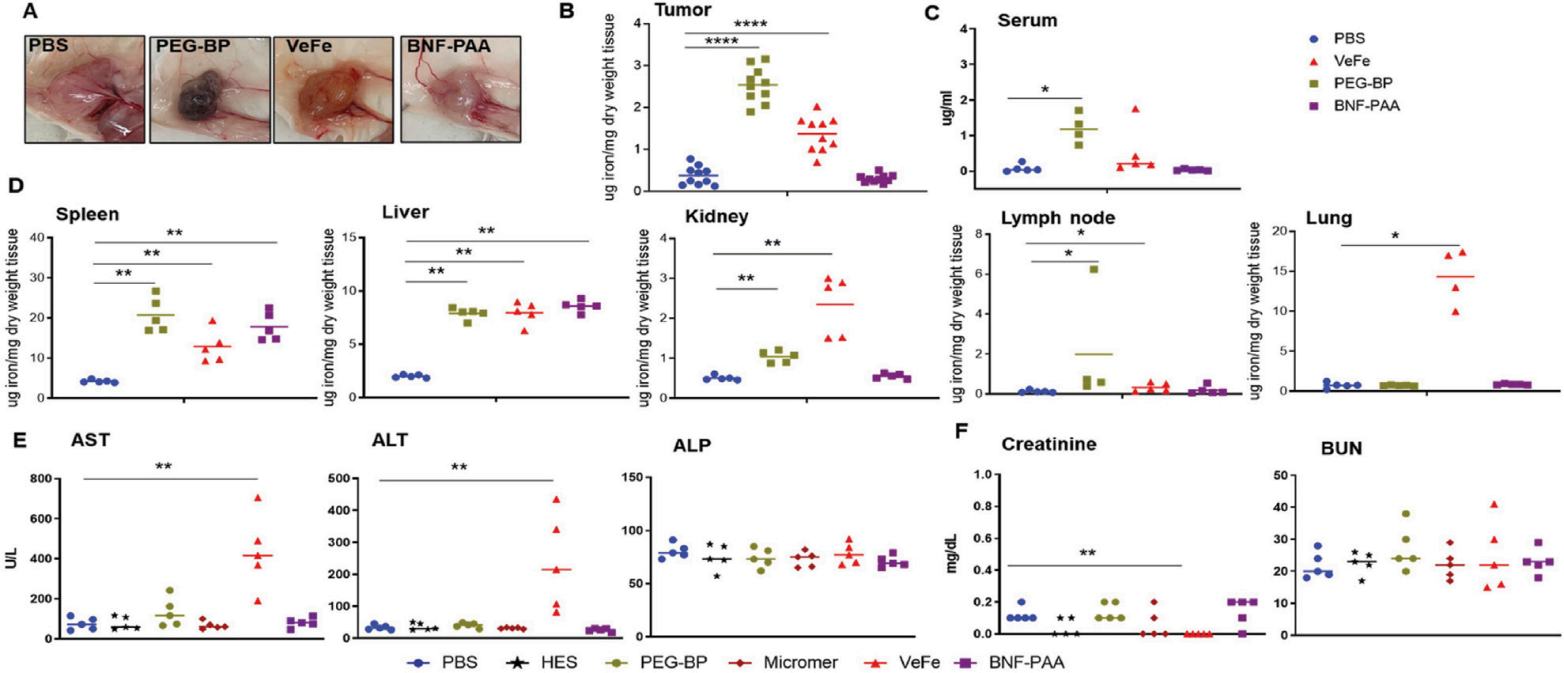
Nanoparticle composition affects iron biodistribution following systemic exposure, and VeFe showed some toxicity. **(A)** Dark coloration due to the presence of nanoparticles was observed in the tumors of mice treated with VeFe and PEG-BP. **(B)** ICP-MS analysis of tumors indicated a significantly increased accumulation of iron that corroborated with visual tumor analysis. **(C, D)** ICP-MS analysis of the serum and other tissues showed the organ- and type of nanoparticle-specific accumulation of iron compared to that in the control. **(E, F)** Liver and kidney function analysis using serum biochemical markers showed elevated levels of liver function enzymes in the VeFe-treated group. Significantly lower creatinine was detected in VeFe-treated mice than in the control (Mann–Whitney test; *p < 0.05, **p < 0.01, and ****p < 0.0001).

The overall study design for evaluating the biodistribution of iron oxide nanoparticles is shown in Figure 1B. Visual analysis of tumors after 24 h of treatment showed dark coloration in the VeFe and PEG-BP cohorts, indicating higher iron accumulation in these tumors compared to the PBS or BNF–PAA cohorts (Figure 5A). Corroborating this, ICP-MS analysis showed significantly higher iron accumulation in the VeFe- and PEG-BP-treated groups than in the PBS-treated group. BNF–PAA-treated animal tumors showed no significant difference in iron content (Figure 5B). Further analysis of the serum and organs also showed distinct iron accumulation in certain organs. Only PEG-BP-treated mice had significantly higher iron content after 24 h of injection in the serum (Figure 5C). The spleen and liver were the primary organs of iron deposition for all iron oxide nanoparticles. PEG-BP and VeFe accumulated significantly in the kidney and lymph node. The VeFe-treated group had significantly higher iron accumulation in the lungs (Figure 5D). We also stained tissue slides with Prussian blue to visualize iron oxide nanoparticle accumulation. Apart from the liver, spleen, lungs, lymph nodes, tumor, and kidneys, we also analyzed other organs such as the heart, adrenal gland, intestine, and bone marrow for iron accumulation (Supplementary Figures S10–S12). Bone marrow showed some accumulation of iron oxide nanoparticles. VeFe showed accumulation of nanoparticles in the lungs. VeFe and PEG-BP also accumulated in the adrenal glands. PEG-BP was comparatively higher in the intestine.

These results show that, except for the spleen and liver, which are macrophage-filled organs, there is an order of preference for the accumulation of iron oxide nanoparticles depending on their coating in other organs. The PEG coating of PEG-BP increased their circulation time, and hence, it was detectable in the serum even after 24 h of injection. Some amount was also detected in the kidney. On the other hand, VeFe showed higher accumulation in the lung, lymph nodes, and kidney, which was likely due to its size (∼12 nm) or sucrose conjugation. Interestingly, BNF-PAA was not accumulated in any of the tested organs or tumors other than the spleen, liver, and kidneys.

Biochemical analysis of the liver and kidney function enzymes showed significantly higher aspartate and alanine transaminases in the serum of VeFe-treated mice (Figure 5E), which indicates some liver damage within 24 h of injection. No other nanoparticle showed any change in the liver function enzymes. Although a significant reduction in creatinine was detected in VeFe-treated mice, it does not indicate any functional changes in the kidney. Kidney toxicity is usually indicated by higher amounts of creatinine or BUN. Control particles, HES, and Micromer^®^ did not show any change for any of the parameters that were tested.

#### 3.5.1 Minimal changes in the whole-blood parameters of tumor-bearing mice with nanoparticles

**FIGURE 6:**
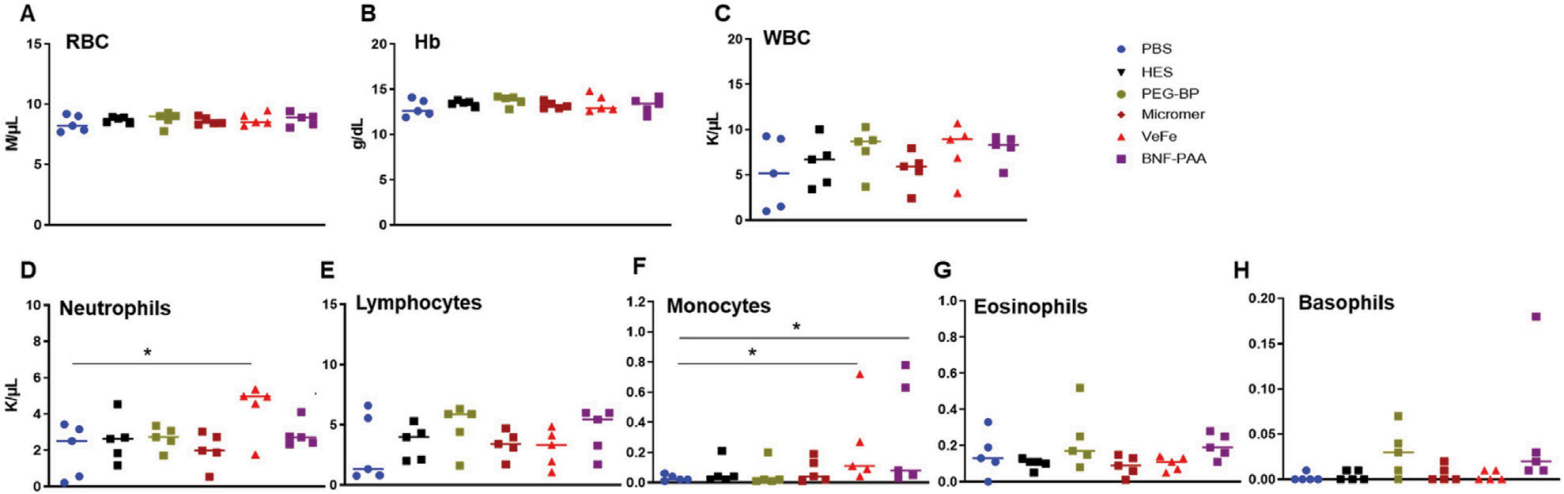
Systemic exposure to nanoparticles induces modest effects on some immune cell lineages, mostly with VeFe nanoparticles. Complete blood count (CBC) of tumor-bearing mice 24 h after injection induced slight variations in neutrophils and monocytes. **(A, B)** RBCs and Hb showed no difference. **(C)** WBC counts showed no difference. **(D)** Neutrophils counts were significantly increased after treatment with VeFe. **(E)** Counts of lymphocytes showed no significant difference. **(F)** Monocyte counts were significantly higher in the VeFe and BNF–PAA cohorts. **(G, H)** Eosinophil and basophils showed no significant change compared to that in the control (Mann–Whitney test; *p = 0.05).

We also analyzed the whole blood of nanoparticle-injected mice after 24 h, as shown in Figures 6A–H. There was no significant difference between any treatment groups in RBCs, Hb, or WBCs (Figures 6A–C). A significant increase in the total number of neutrophils was detected in the VeFe-treated group of mice (Figure 6D). The circulating monocyte population was significantly increased in the VeFe- and BNF–PAA-treated groups. No other parameters showed any change. As shown before, both HES and Micromer^®^ did not show any effect and served as controls.

## 4 Discussion

Many studies have reported immunological perturbances associated with metallic nanoparticles administered via the tail vein in mouse models (Park et al., 2016; Mohammadpour et al., 2020; Brown et al., 2018; Yang et al., 2017; Wang et al., 2016). Simultaneously, researchers are exploring how a better understanding of the effects of nanoparticles on the immune system can be leveraged to harness the immune system to curb disease progression (Perciani et al., 2021). Iron oxide nanoparticles are reported to have an immune-modulating effect that can inhibit tumor progression (Soetaert et al., 2020; Canese et al., 2021; Cheng et al., 2021). In this study, we evaluated the effect of iron oxide nanoparticle formulations with different coatings, including FDA-approved iron nanoparticle (Venofer), and compared their effect on various biochemical and immunological parameters after a single dose of intravenous injection in healthy and tumor-bearing mice. Overall, no adverse effect was noted with any of the formulations for the 60-day observation period. No weight loss was observed, and all the mice were healthy and gained weight over time. Upon sacrifice after 60 days, blood biochemistry also showed no indication of toxicity in the liver or kidney. A transient effect on the immune cells was observed, but most of those changes were within the normal range. There was an increase in the number of liver inflammatory foci in the iron oxide nanoparticle-treated groups. This can be attributed to the normal process for digesting accumulated iron oxide particles as no microscopic damage was observed in the liver tissue. Based on their coating, there were some differences in organ distribution (such as VeFe in the lungs), but largely, most of the nanoparticles accumulated in the liver and spleen. It is noteworthy that Micromer^®^, the HES-coated non-magnetic nanoparticles, had the least effect on peripheral blood cells among the nanoparticles, which shows that the core of the nanoparticle can influence the outcome in mice. Venofer, which was developed as a fast-releasing iron compound for the treatment of iron-deficiency anemia (IDA), had comparatively greater effect on all parameters than slow iron-releasing nanoparticles such as PEG-BP and BNF–PAA. This might be because we used more than the recommended dosage (Helenek et al., 2023) for this formulation to compare it with slow iron-releasing nanoparticles. In tumor-bearing mice, PEG-BP accumulated in tumors in contrast to VeFe or BNF–PAA. Hence, it can be concluded that the coating possibly influences the nanoparticle accumulation in tumors more than enhanced permeability and retention (EPR).

Significant accumulation of PEG-BP in tumors may enable their use as MRI contrast agents for the detection of tumors. Though VeFe has not been tested as an MRI contrast agent, several pilot clinical studies have used the FDA-approved iron oxide nanoparticle, ferumoxytol, as an MRI contrast agent for tumor diagnosis (Toth et al., 2017). Future studies focusing on exploring PEG-BP as a potential MRI contrast agent for diagnostic purposes are needed. We also noticed that after 24 h, PEG-BP was still present in circulation, as evident from the serum ICP-MS. Even after 60 days, the major organs of the reticuloendothelial system, the liver and spleen, had substantial accumulation of iron oxide nanoparticles. Although we have not tested whether there are any long-term consequences because of the iron load, it is well-known that clinically approved iron oxide nanoparticles, including VeFe, accumulate in the liver and release iron over time to mitigate anemia (Qunibi, 2010; Macdougall et al., 2020; Geisser and Burckhardt, 2011). Venofer has been used clinically since 1949 and has been proven safe in patients for decades (Qunibi, 2010). For some of the newer FDA-approved, more complex iron oxide nanoparticle formulations for anemia, the recommended injection dosage is substantially higher and needs only a single administration. This is because they release iron over a very long time, and hence, the organ clearance can take months to years, so the patient does not need to visit the clinic often (Friedrisch and Cançado, 2015; Van Doren et al., 2024). Moreover, in clinical settings, excess iron after treating iron-deficiency anemia, transfusion overload of iron in thalassemia, and excessive iron absorption in secondary hemochromatosis are managed through iron chelator therapy (Entezari et al., 2022; Salimi et al., 2024). Understanding the risk and the vast knowledge of mitigating iron overload in patients will enable the safe use of iron oxide nanoparticles in oncological settings.

This study was restricted to HER2-positive breast cancer as a model for toxicity and biodistribution analysis. The iron oxide nanoparticles we used in this study are devoid of any targeting moiety, and hence, they can be adapted to other cancer models. Further studies are warranted in other cancer models, such as pancreatic and triple-negative breast cancers, to explore the versatility of nanoparticle distribution in those aggressive cancer types that need more treatment options in combination with the standard of care. In conclusion, this study shows that iron oxide nanoparticles exert a direct, yet reversible, effect on immune cells, with no acute or chronic adverse effects observed in any organs at a human-equivalent dose. Thus, these nanoparticles can be further evaluated for their immunomodulatory effect in the context of disease.

## Data Availability

The original contributions presented in the study are included in the article/Supplementary Material; further inquiries can be directed to the corresponding authors.
