# Supplementary material for: Intravenously administered iron oxide nanoparticles with different coatings reversibly perturb immune cells in peripheral blood without inducing toxicity in mice
Source: Front Toxicol. 2025 Oct 14;7:1673416. doi: 10.3389/ftox.2025.1673416 (PMC12558996; doi:10.3389/ftox.2025.1673416)
Supplement: Supplementary file 1 [file Table1.docx]

**Table S1**

**Physical characteristics of nanoparticles**

| **Nanoparticles** | **Lot** | **Z-Average (nm)** | **PDI** | **ZP** | **pH** |
| --- | --- | --- | --- | --- | --- |
| **PEG-BP** | 1401910-01 | 174 | 0.129 | -16.5 | 7.6 |
| **BNF-PAA** | 0771483-22 | 131 | 0.109 | -28.1 | 7.4 |
| **micromer^®^** | 1911910-01 | 117 | 0.021 | -41.6 | 7.1 |
