# Supplementary material for: Intravenously administered iron oxide nanoparticles with different coatings reversibly perturb immune cells in peripheral blood without inducing toxicity in mice
Source: Front Toxicol. 2025 Oct 14;7:1673416. doi: 10.3389/ftox.2025.1673416 (PMC12558996; doi:10.3389/ftox.2025.1673416)
Supplement: Supplementary file 2 [file DataSheet1.pdf]

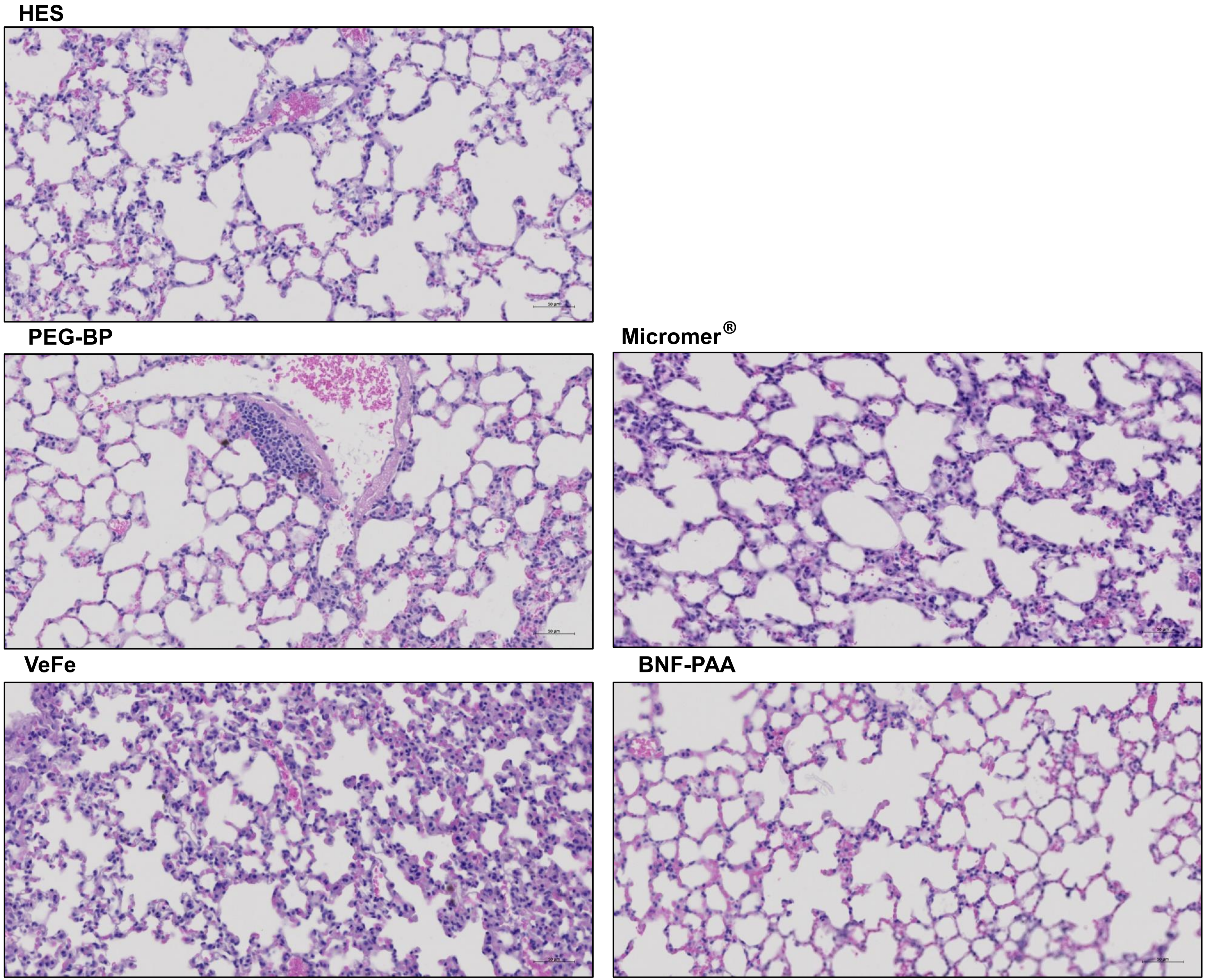

Figure S1: Representative H&E image of lung from each treatment group shows no signs of toxicity

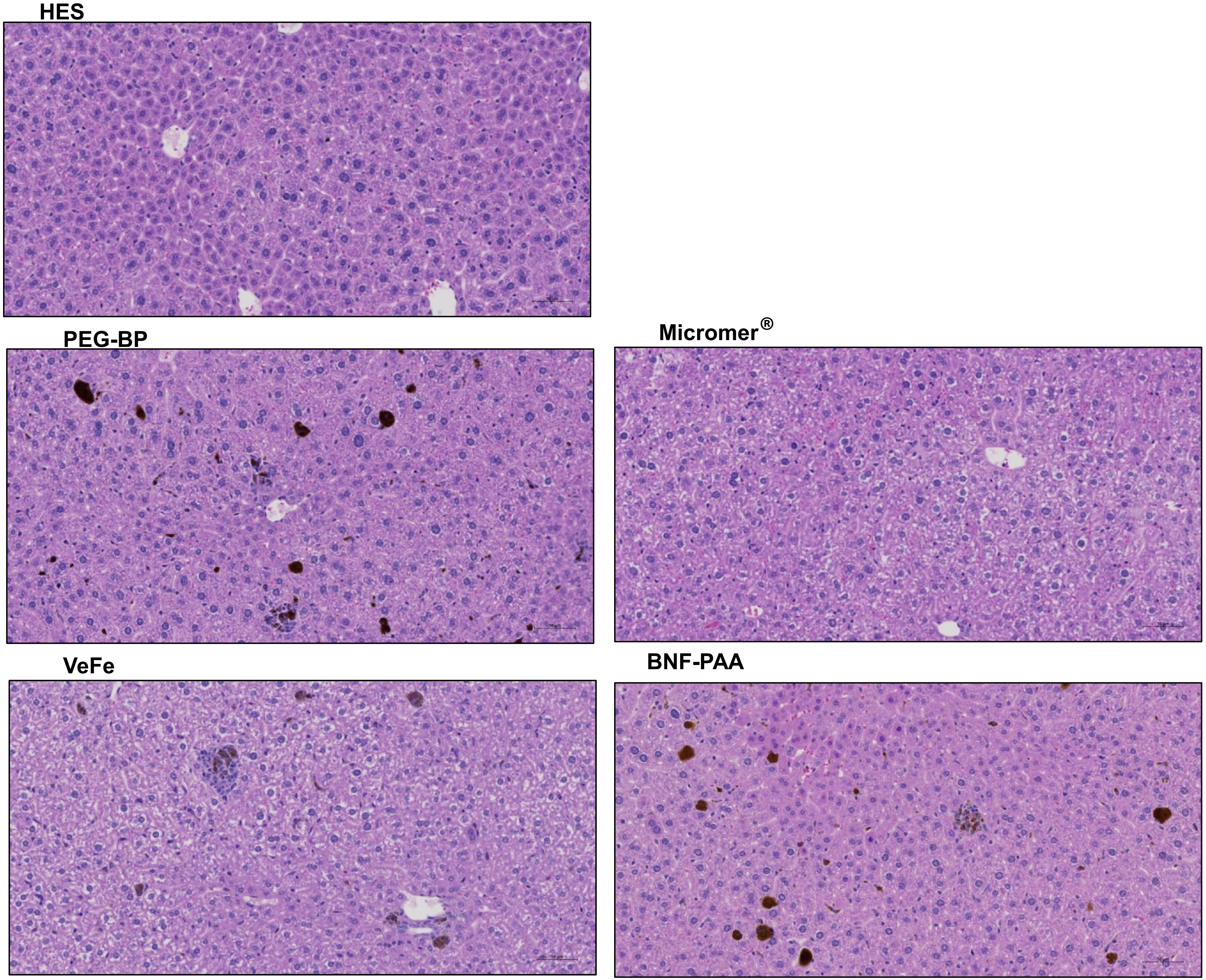

Figure S2: Representative H&E image of liver from each treatment group shows no signs of toxicity

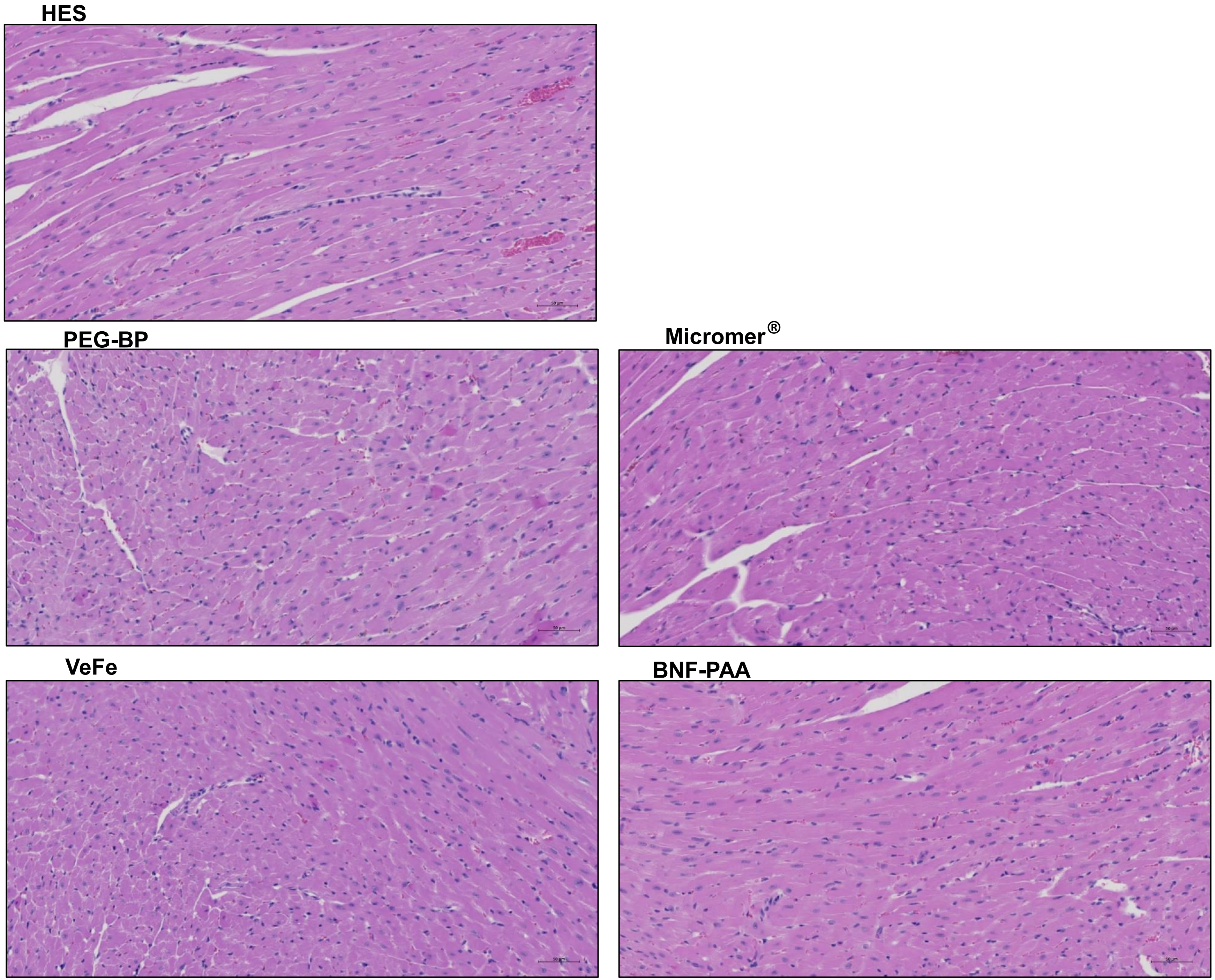

Figure S3: Representative H&E image of heart from each treatment group shows no signs of toxicity

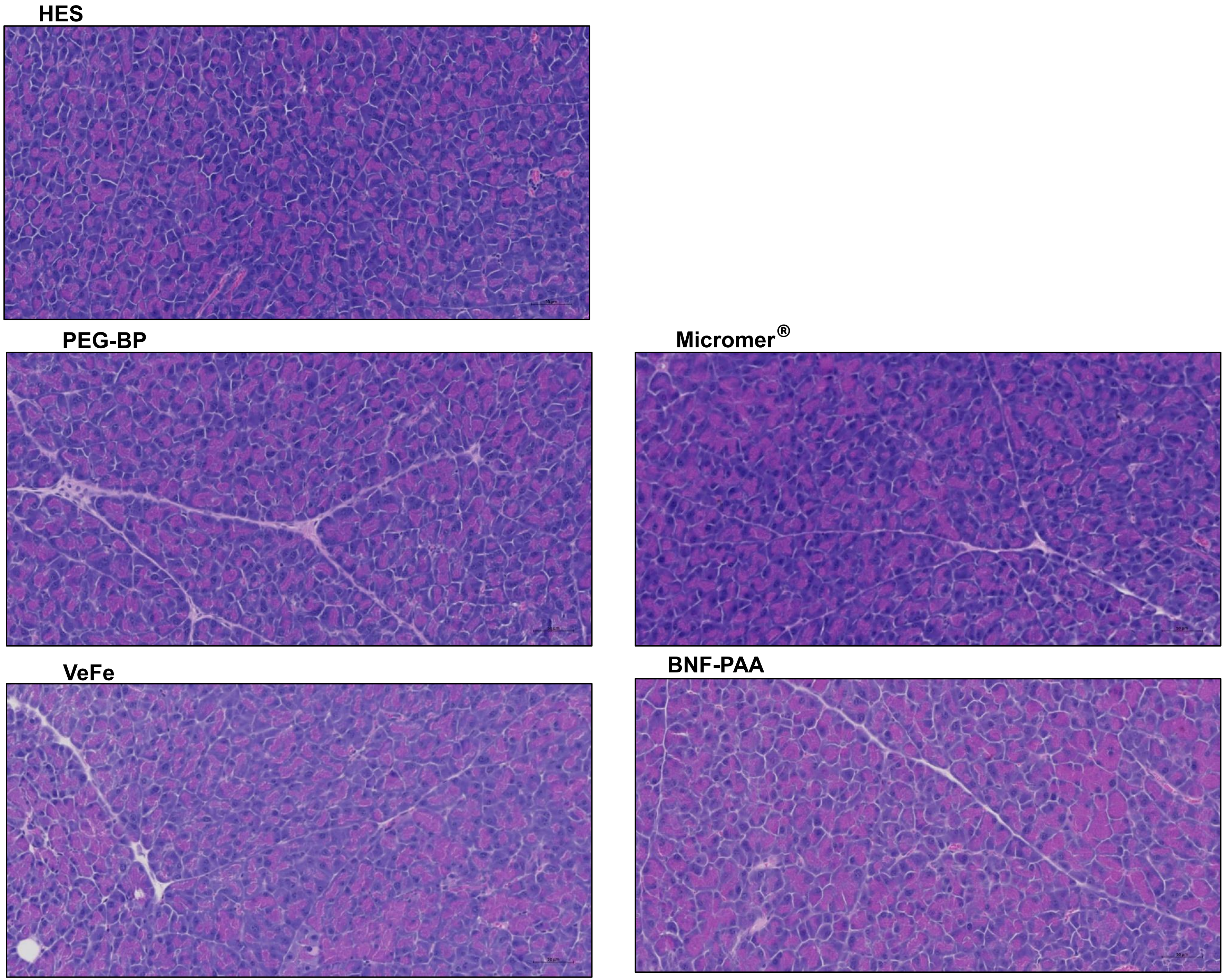

Figure S4: Representative H&E image of Pancreas from each treatment group shows no signs of toxicity

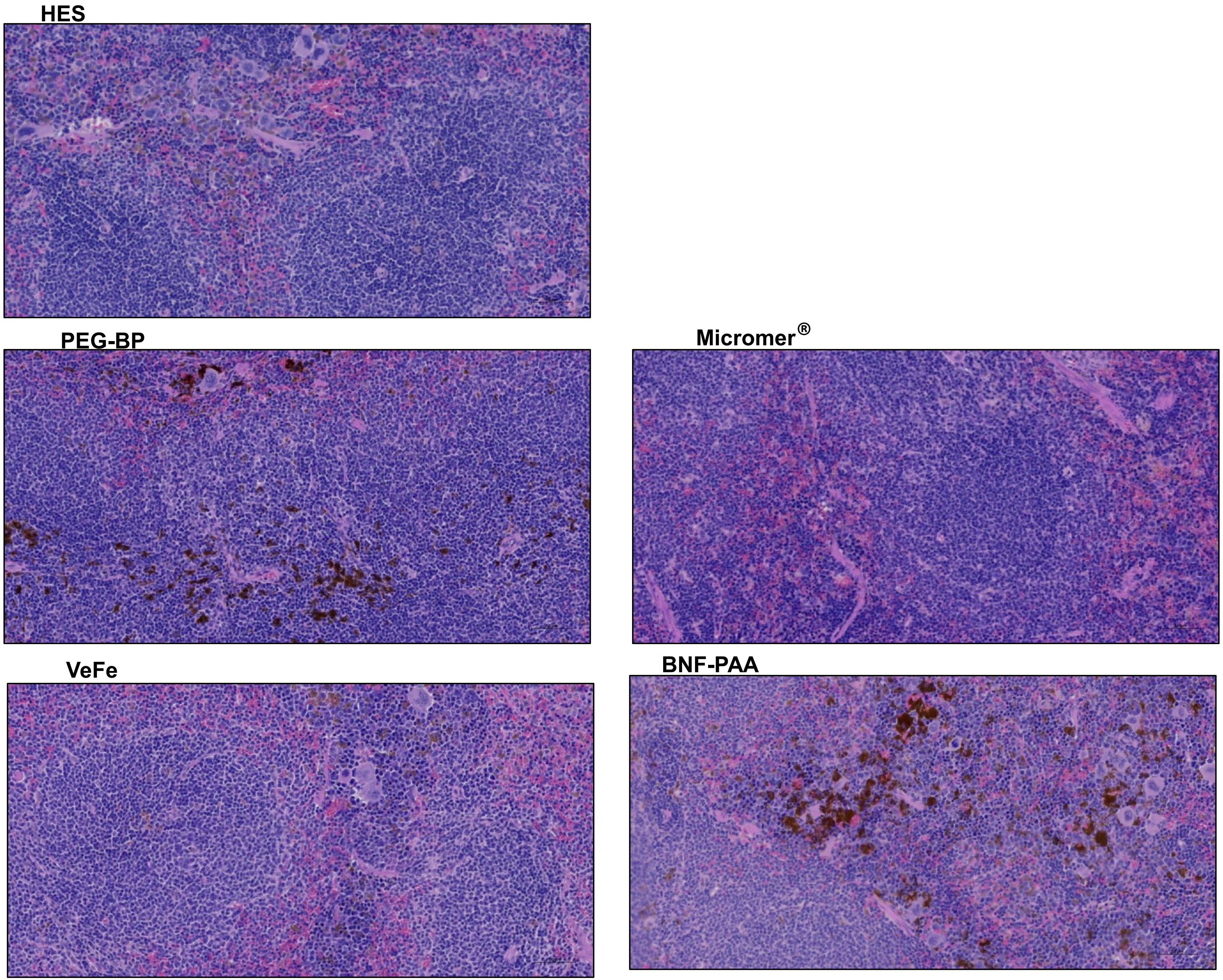

Figure S5: Representative H&E image of Spleen from each treatment group shows no signs of toxicity

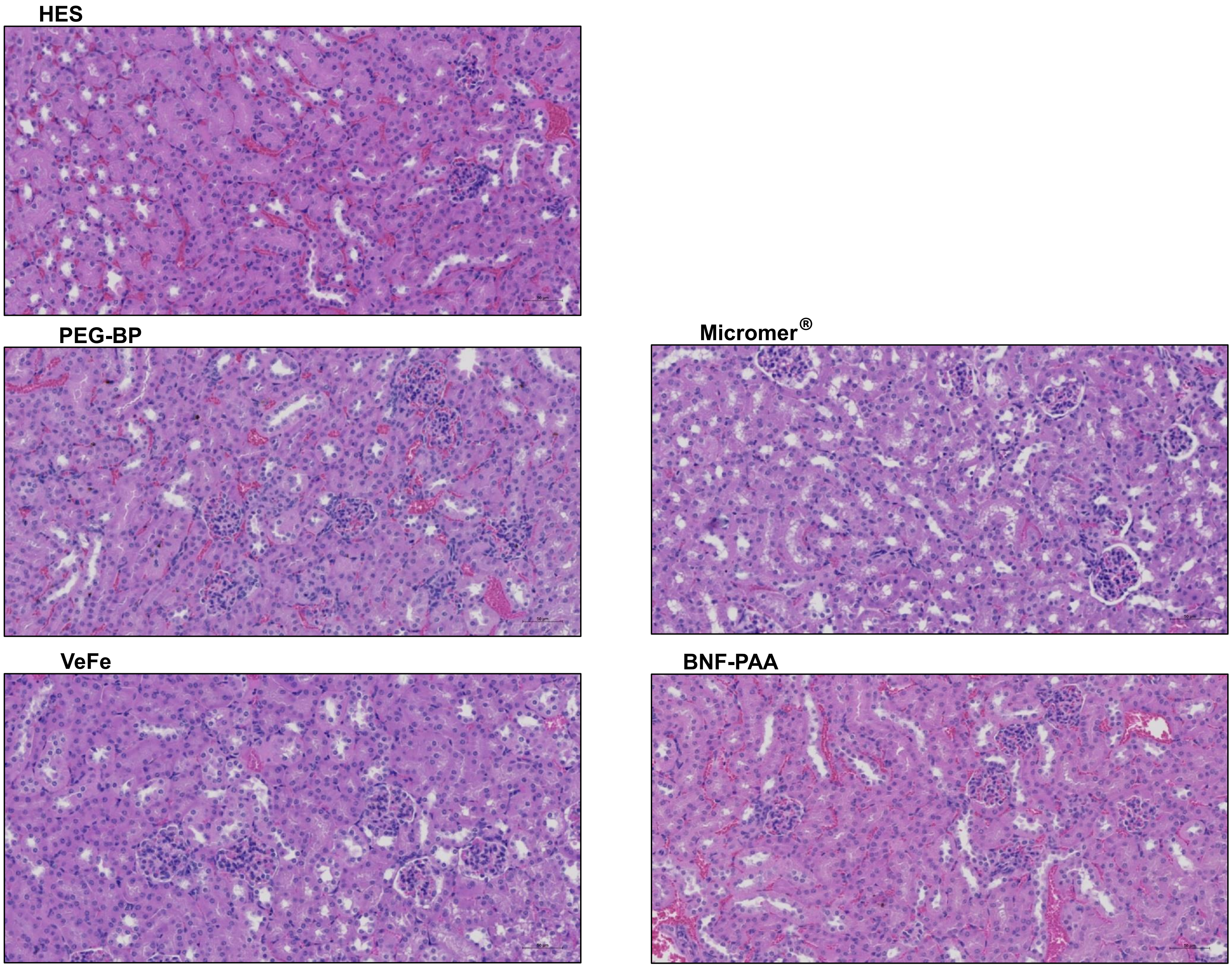

Figure S6: Representative H&E image of kidney from each treatment group shows no signs of toxicity

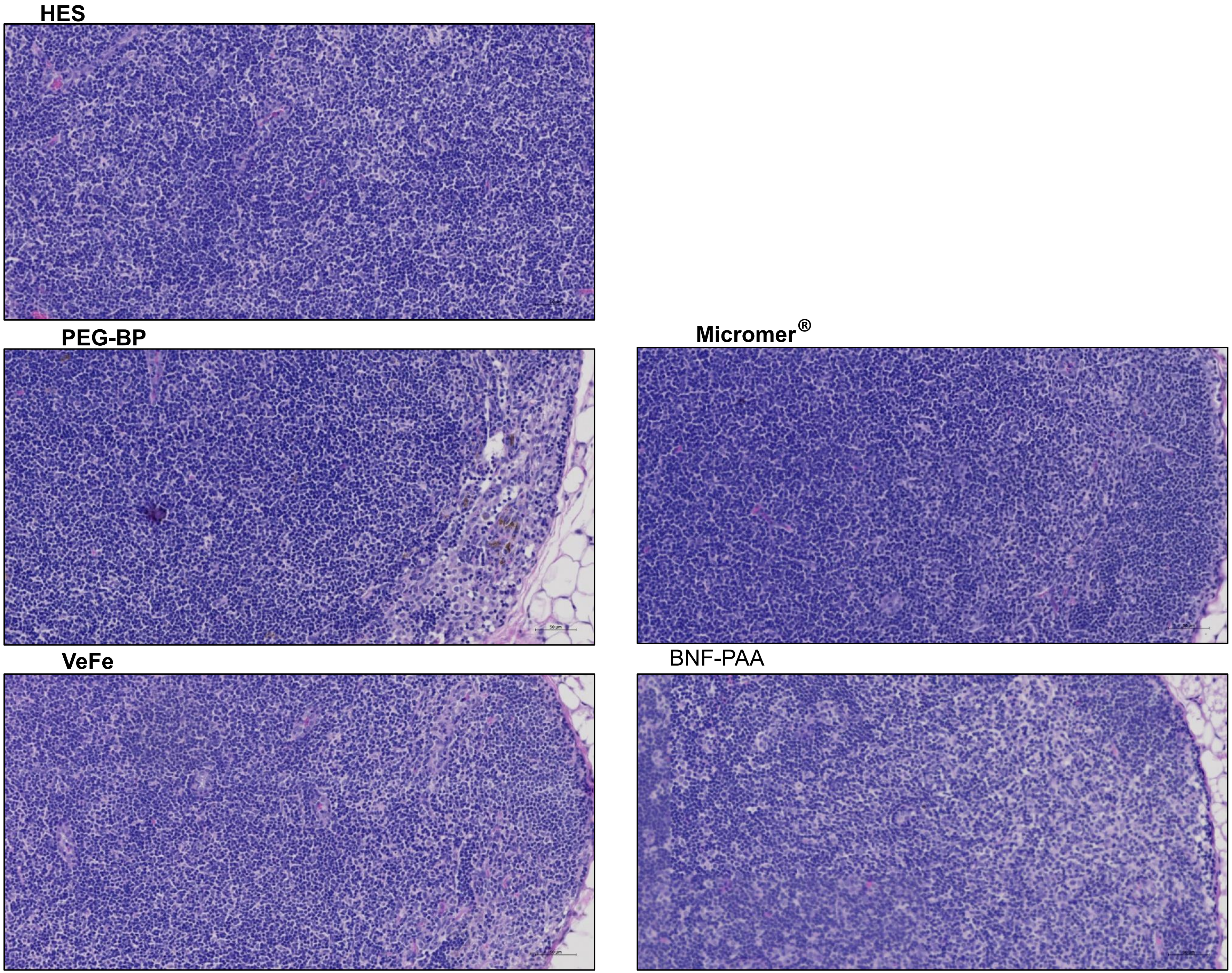

Figure S7: Representative H&E image of lymph node from each treatment group shows no signs of toxicity

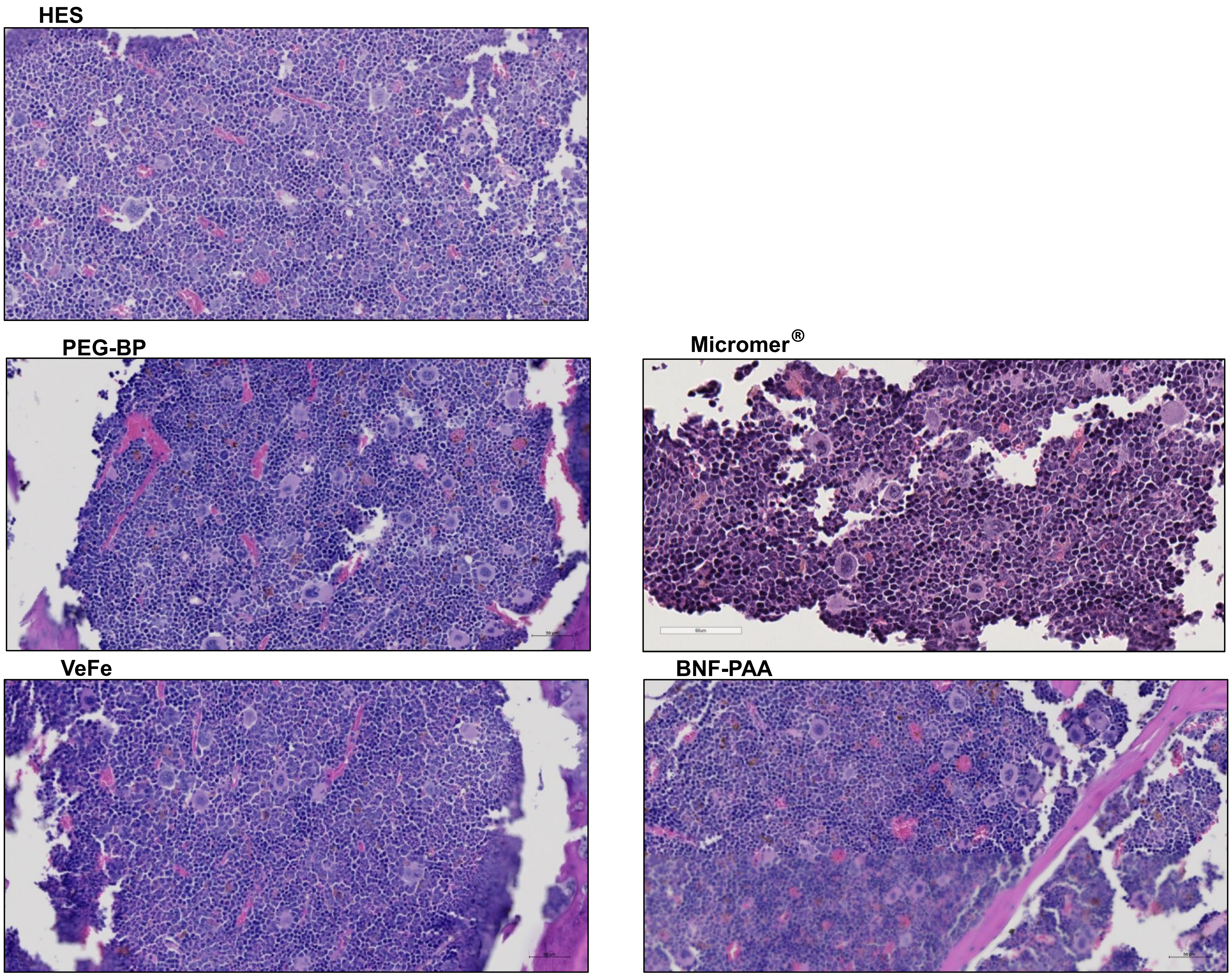

Figure S8: Representative H&E image of bone marrow from each treatment group shows no signs of toxicity

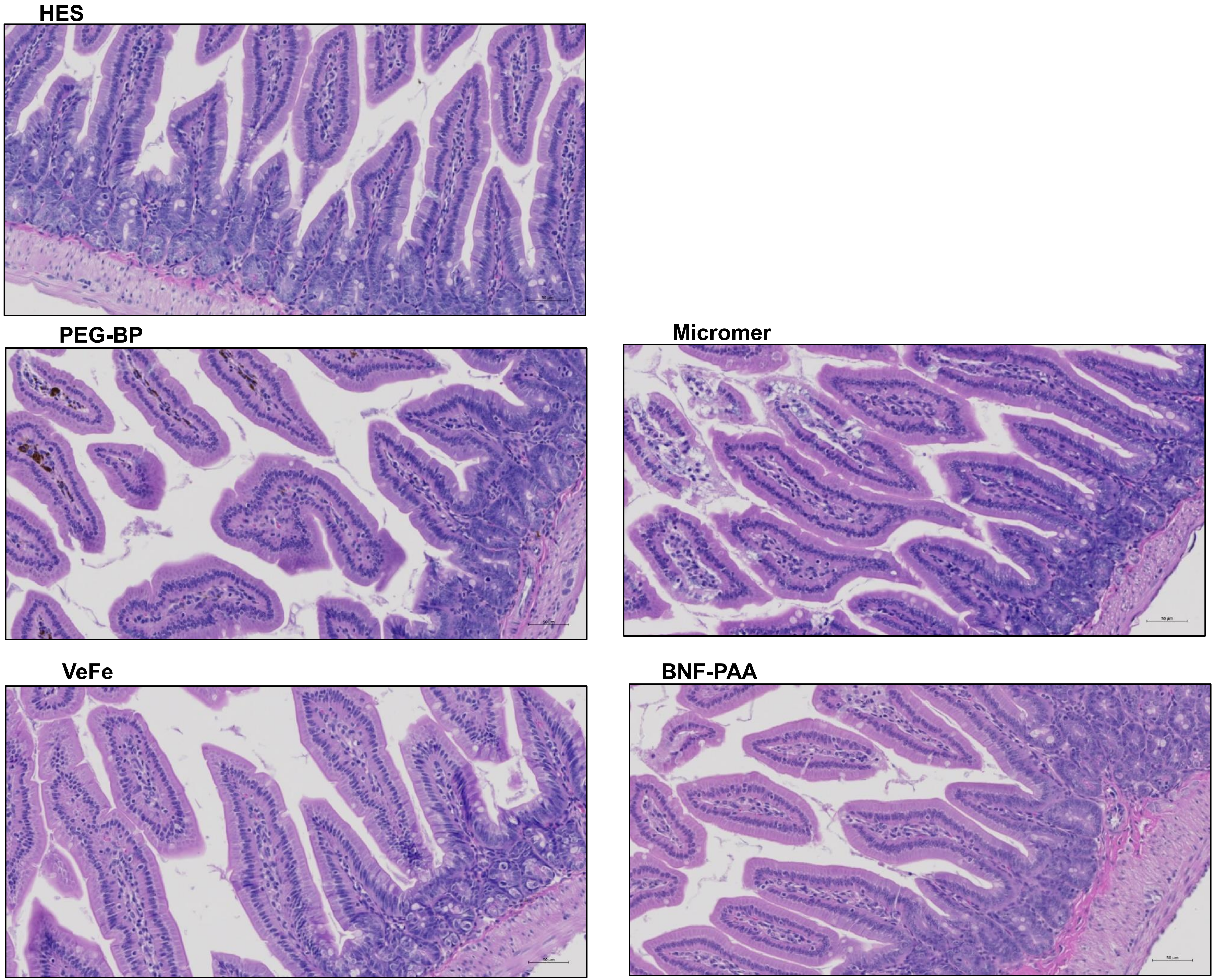

Figure S9: Representative H&E image of intestine from each treatment group shows no signs of toxicity

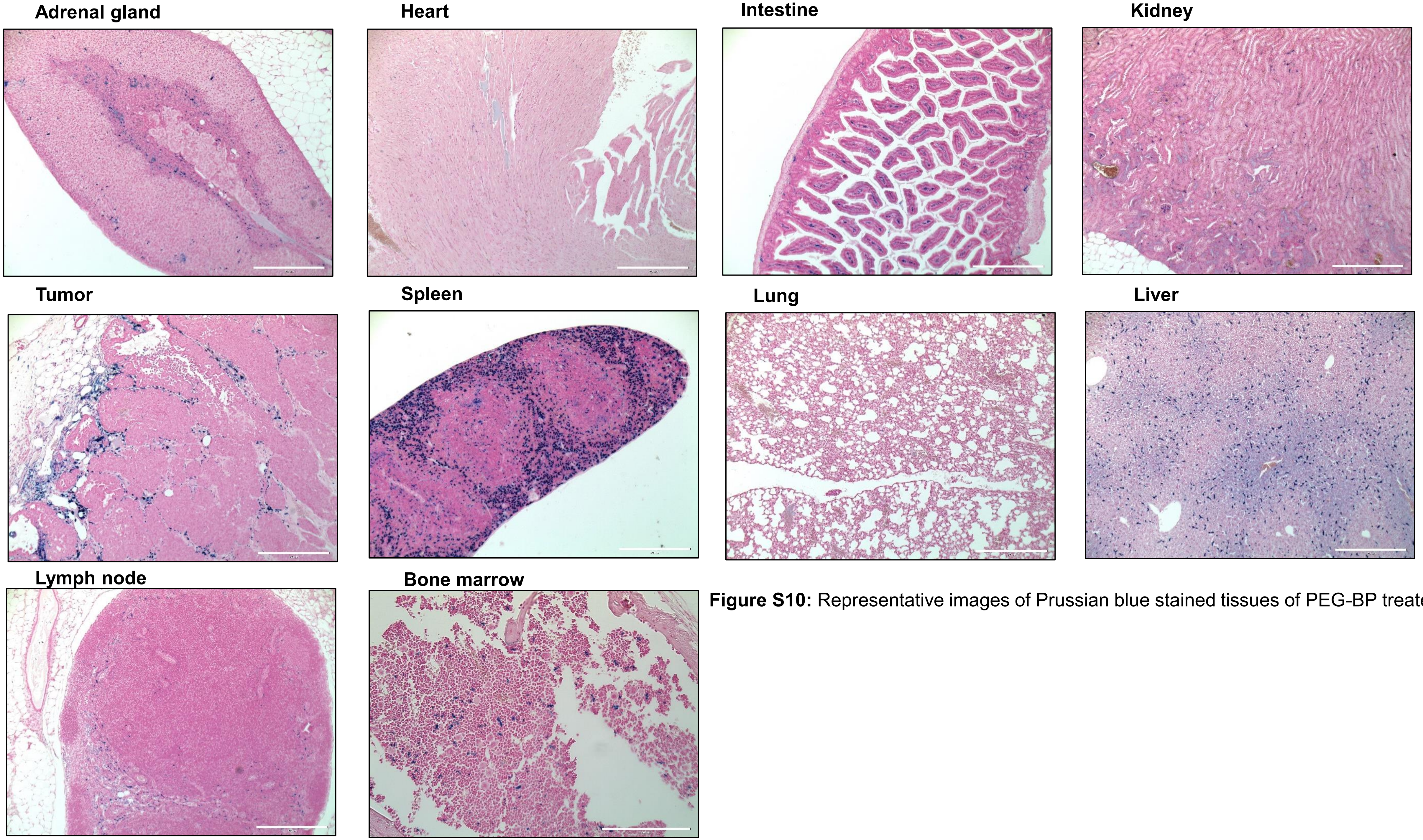

Figure S10: Representative images of Prussian blue stained tissues of PEG-BP treated group

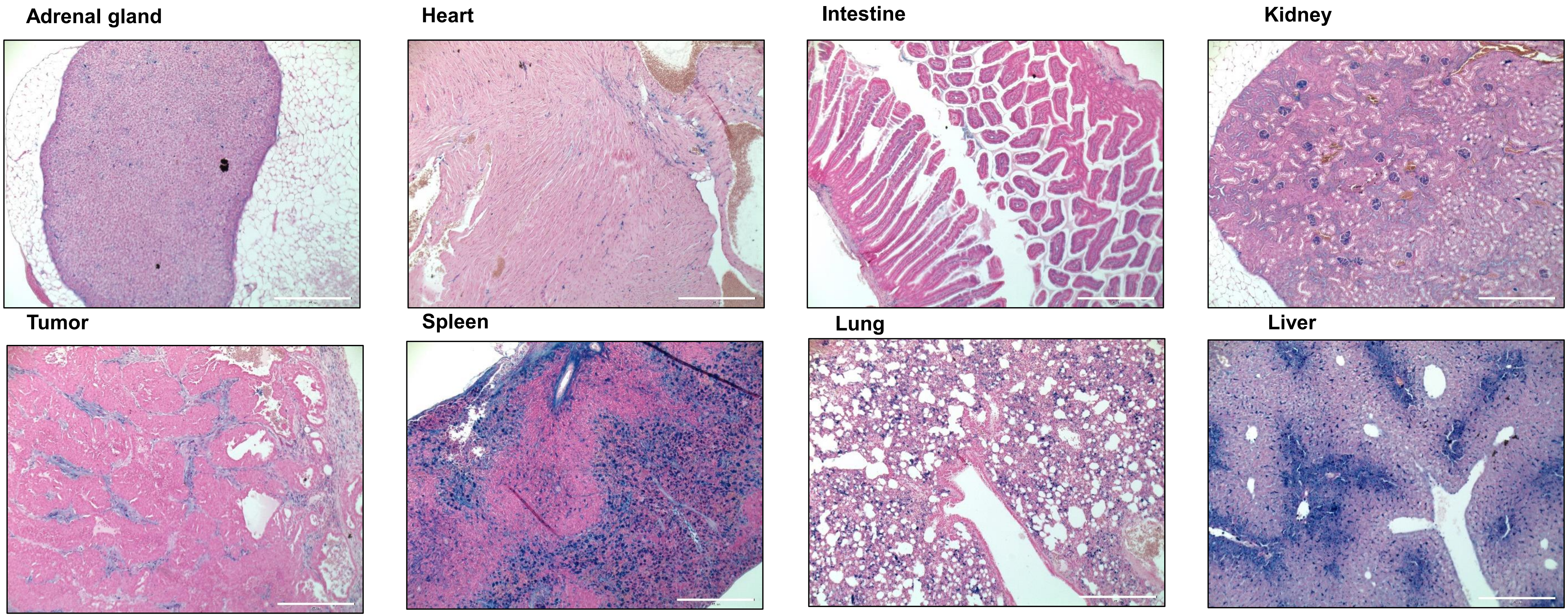

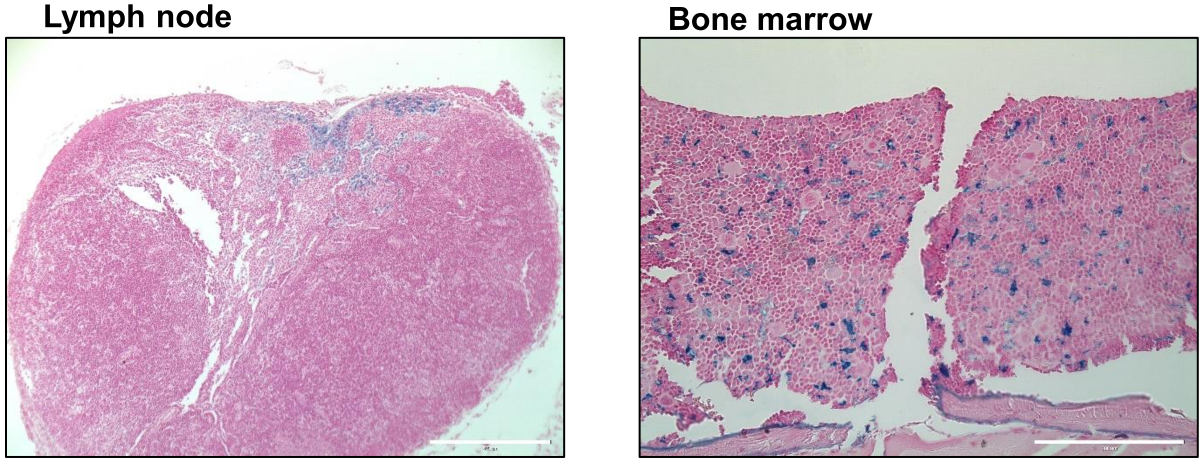

Figure S11: Representative images of Prussian blue stained tissues of VeFe treated group

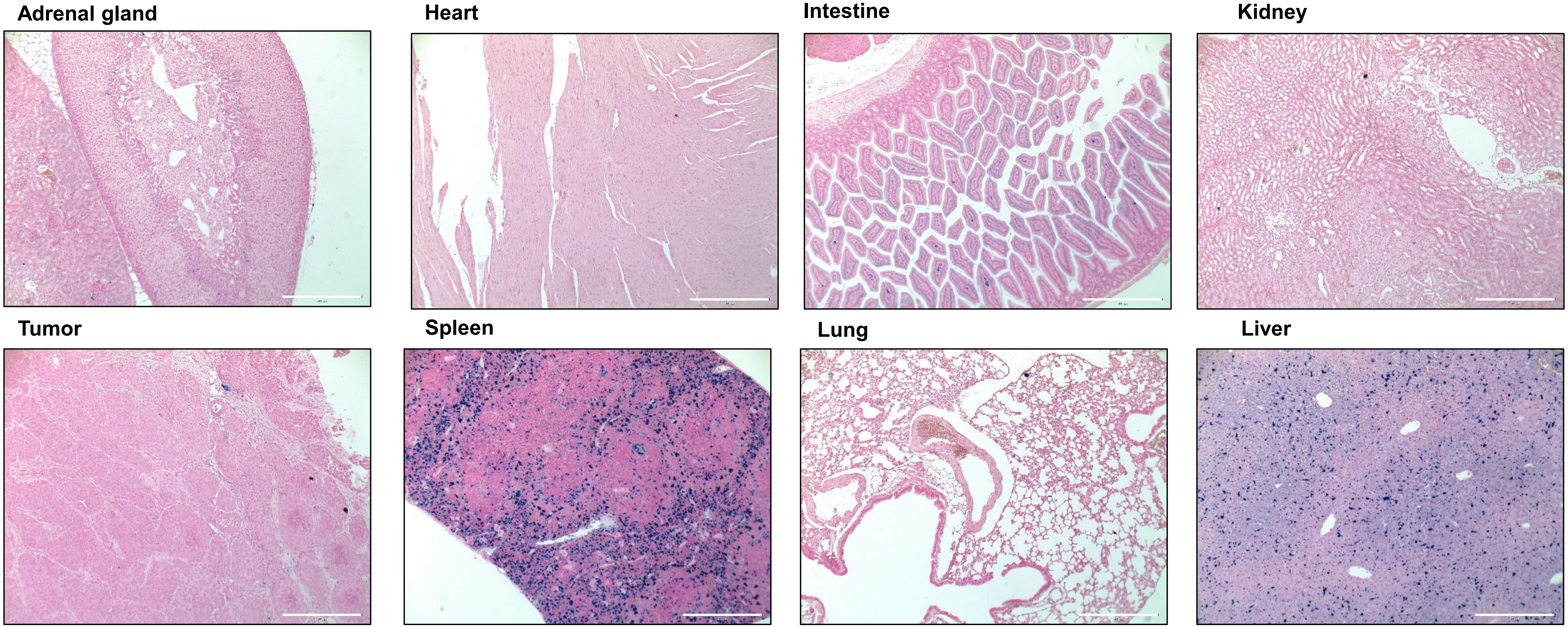

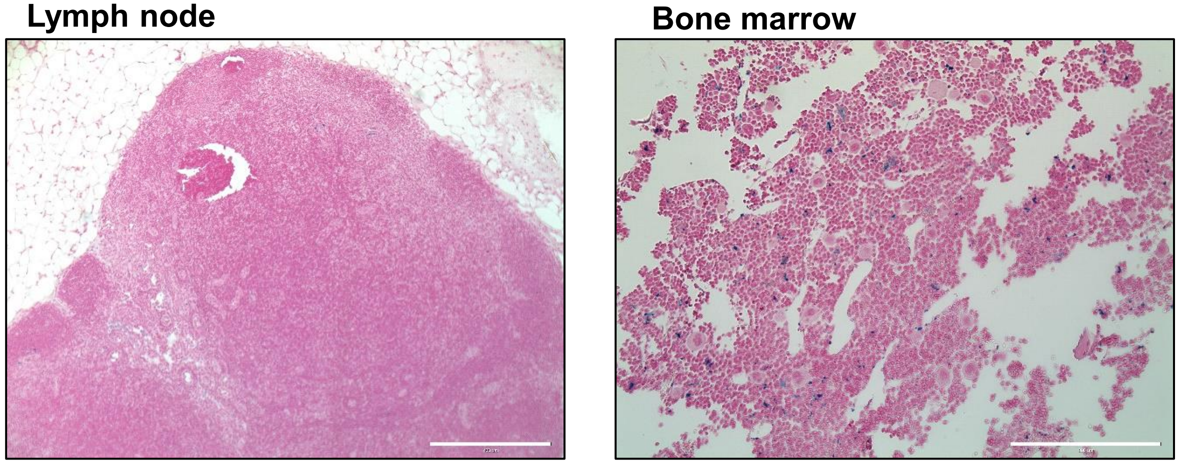

Figure S12: Representative images of Prussian blue stained tissues of BNF-PAA treated group
